# Characteristics and natural course of vertebral endplate signal (Modic) changes in the Danish general population

**DOI:** 10.1186/1471-2474-10-81

**Published:** 2009-07-03

**Authors:** Tue S Jensen, Tom Bendix, Joan S Sorensen, Claus Manniche, Lars Korsholm, Per Kjaer

**Affiliations:** 1 The Back Research Center, Ringe, Denmark; 2 Institute of Sports Science and Clinical Biomechanics, University of Southern Denmark, Odense, Denmark; 3 Department of Statistics, University of Southern Denmark, Odense, Denmark

## Abstract

**Background:**

Vertebral endplate signal changes (VESC) are more common among patients with low back pain (LBP) and/or sciatica than in people who are not seeking care for back pain. The distribution and characteristics of VESC have been described in people from clinical and non-clinical populations. However, while the clinical course of VESC has been studied in patients, the natural course in the general population has not been reported. The objectives of this prospective observational study were to describe: 1) the distribution and characteristics of VESC in the lumbar spine, 2) its association with disc degeneration, and 3) its natural course from 40 to 44 years of age.

**Methods:**

Three-hundred-and-forty-four individuals (161 men and 183 women) sampled from the Danish general population had MRI at the age of 40 and again at the age of 44. The following MRI findings were evaluated using standardised evaluation protocols: type, location, and size of VESC, disc signal, and disc height. Characteristics and distribution of VESC were analysed by frequency tables. The association between VESC and disc degeneration was analysed by logistic regression analysis. The change in type and size of VESC was analysed by cross-tabulations of variables obtained at age 40 and 44 and tested using McNemar's test of symmetry.

**Results:**

Two-thirds (67%) of VESC found in this study were located in the lower part of the spine (L4-S1). VESC located at disc levels L1-L3 were generally small and located only in the anterior part of the vertebra, whereas those located at disc levels L4-S1 were more likely to extend further into the vertebra and along the endplate. Moreover, the more the VESC extended into the vertebra, the more likely it was that the adjacent disc was degenerated. The prevalence of endplate levels with VESC increased significantly from 6% to 9% from age 40 to 44. Again, VESC that was only observed in the endplate was more likely to come and go over the four-year period compared with those which extended further into the vertebra, where it generally persisted.

**Conclusion:**

The prevalence of VESC increased significantly over the four-year period. Furthermore, the results from this study indicate that the distribution of VESC, its association with disc degeneration and its natural course, is dependent on the size of the signal changes.

## Background

Vertebral endplate signal changes (VESC) have been proposed to be a cause of low back pain (LBP)[1,2]. In a recent systematic literature review, the prevalence of VESC was found to increase with age and to be lower in non-clinical populations (i.e. non-LBP, general, and working populations) than in clinical populations (people with LBP and/or sciatica), with median prevalence rates of 6% and 43% respectively. Moreover, a statistically significant positive association between VESC and LBP was found in 7 out of 10 articles that reported sufficient data to calculate odds ratios[3].

In a descriptive study of VESC, Modic et al investigated 474 patients referred for lumbar Magnetic Resonance Imaging (MRI)[4]. They described two types of signal changes: type 1 seen as hypointensity on T1-weighted images and hyperintensity on T2, and type 2 seen as hyperintensity on T1 and T2-weighted images. Further histological examination of type 1 changes in three patients, showed fissured endplates and vascular granulation tissue adjacent to the endplates. In three patients with type 2 changes, histology also identified disruption of the endplates as well as fatty degeneration of the adjacent bone marrow[4]. Later, type 3 was described as hypointensity on T1 and T2-weighted images representing sclerosis as seen on radiographs[5].

The distribution and characteristics of VESC in the lumbar spine have been described in patients with non-specific LBP and/or sciatica [4,6-9], in the working population[10,11], and in people without LBP[12]. In people who have no LBP, VESC has been described to be focal and located in the anterior third of the superior endplates of the mid lumbar vertebrae[12]. In contrast, in people seeking care for LBP and/or sciatica, VESC has been described to be distributed equally between the superior and inferior endplates, to have a larger extent, and to be more prevalent in the lower lumbar spine than in the upper lumbar spine[8,9]. Several studies have reported that VESC is often seen adjacent to degenerated or herniated discs[4,6,8-10,13-15]. In fact, the term 'Modic changes' is described as the combination of VESC and disc degeneration[4]. However, in a study of 59 individuals without LBP, there was no correlation between focal type 1 changes and disc degeneration[12].

The natural course of VESC has been investigated in longitudinal studies of patients with LBP and/or sciatica[4,6,9,16] and in people with no LBP[17]. From these studies, VESC seems to be stable in 48% to 86% of people and to convert from one type to another in 14% to 52% over periods of 14 to 72 months. In three of the five studies, new VESC appeared over time in 6% to 34% of levels/individuals without VESC at baseline[6,9,17]. In one study of 166 patients with sciatica, the signal changes disappeared in 16% of 38 patients who had VESC at baseline[6].

To our knowledge, the distribution and characteristics of VESC have not been described in the general population. The aims of this study were to describe the distribution and characteristics of VESC in the lumbar spine, its association with disc degeneration, and its natural course from the age of 40 to 44.

## Methods

### Study sample

In this prospective observational study, people sampled from the Danish general population were MRI-scanned at the age of 40 years (in 2000/2001) and 44 years (in 2004/2005). Details of this cohort have been described previously[18]. Permission for the study was granted by the local ethics committee (ref. no. 20000042) and for the database by the Danish Data Protection Agency (ref. no. 2000-53-0037). Informed consent was signed by all participants after they were informed about the study.

### MRI protocol

MRI was performed with a 0.2 T MRI-system (Magnetom Open Viva; Siemens AG, Erlangen, Germany). A body spine surface coil was used with the participants lying in the supine position. The following sequences were used:

• A localizer sequence of five images, 40/10/40 degrees (TR/TE/flip angle) consisting of two coronal and three sagittal images in orthogonal planes

• Sagittal T1-weighted spin echo, 621/26 (TR/TE), 144 × 256 matrix, 300 mm. field of view, 11 slices of 4 mm. thickness, 2 acquisitions, 6 min. 1 sec. scan time

• Sagittal T2-weighted turbo spin echo 4609/134 (TR/effective TE), 210 × 256 matrix, 300 mm. field of view, 11 slices of 4 mm. thickness, 2 acquisitions, 8 min. 42 sec. scan time

• Axial T2-weighted turbo spin echo 6415/134 (TR/effective TE), 180 × 256 matrix, 250 mm field of view, 15 slices of 5 mm. thickness, 2 acquisitions, 7 min. 49 sec. scan time. Slices were placed in the plane of the five lower discs.

### MRI evaluation

The MRI evaluation was performed by an experienced radiologist (JSS), the first author (TSJ) and a second chiropractor (Chiro2) using standardized evaluation protocols[19,20]. JSS evaluated disc signal and disc height for all image sets. TSJ and Chiro2 evaluated the VESC findings idependently, so that TSJ evaluated 58 of the 688 image sets and Chiro2 evaluated the remaining 630 cases.

The inter observer reproducibility of disc height and disc signal between JSS and a second radiologist, has previously been reported to be 0.59 and 0.66, respectively[20]. The training of TSJ and the reproducibility of the evaluation of VESC findings has been previously reported[19]. In this study, MRIs of 50 individuals were evaluated independently by JSS and TSJ. The Kappa value for inter observer reproducibility of the evaluation of VESC per endplate between JSS and TSJ was 0.80.

Chiro2 is a chiropractic radiologist who has been trained in radiology in a three year full-time residency program (DACBR) and in three Fellowships in musculoskeletal radiology and neuroradiology. The training of Chiro2 in this study was carried out by TSJ under supervision of the radiologist. After introduction to the evaluation protocol, 18 cases from the study cohort were read in a joint session in order to reach consensus. After consensus was reached, 38 image sets from the study cohort were evaluated independently by TSJ and Chiro2. The Kappa value for inter-observer reproducibility between the two readers for the 38 cases was 0.51, which was lower than the limit of 0.6 that was predefined as acceptable. Communication on the reasons for disagreement was undertaken and a new round of consensus readings of 8 cases was performed. Nine weeks after the first reproducibility study, the same 38 cases and 12 new cases were evaluated independently by the two chiropractors. The results from the chiropractors evaluations were compared to the radiologists original readings. The Kappa value for inter-observer reproducibility between the two chiropractors and the radiologist was 0.81 which was above the cut-point of 0.6 for having acceptable agreement.

### Variables of interest

For the purpose of the present study, the type, location, and size of VESC were evaluated for each lumbar endplate (L1 – S1). The type of VESC was graded for each lumbar endplate (L1-S1) as either type 1, type 2, or type 3 as described by Modic et al[4]. If more than one type was present within the same endplate, that endplate was graded as a mixed type.

**Figure 1:**
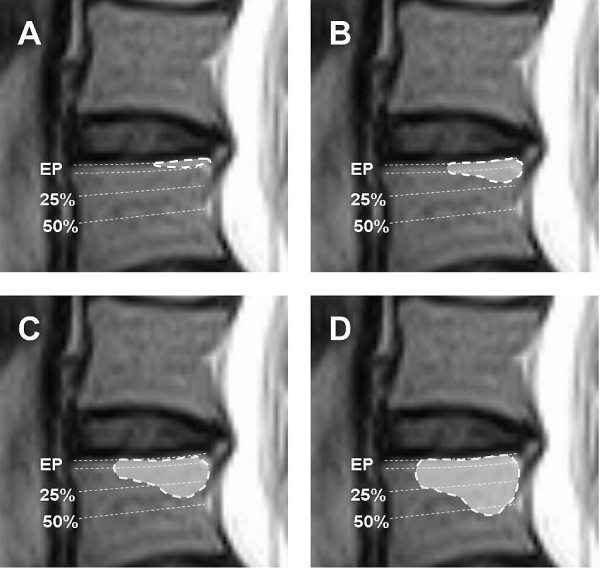
**Size of VESC**. Classification of the size of VESC related to its relative depth of extension of the vertebral body height: **A**: Endplate (EP) only, **B**: < 25%, **C**: 25–50%, and **D**: > 50%[19].

The location of VESC was defined as involving: 1) the central part of the endplate, 2) the anterior part, 3) the posterior part, 4) the lateral parts, or 5) two or more of the previous locations. The size of VESC was defined as the maximum cranio-caudal extension: 0 = No VESC, 1 = observed in the endplate (EP) only, 2 = <25% of vertebral body height, 3 = 25% to 50% of vertebral body height, and 4 = >50% of vertebral body height as seen on the sagittal images (see Figure 1).

Disc degeneration was defined as a hypointense disc (grade 3)[21,22] or reduced disc height (grades 2 or 3) [22-24].

### Validity of variables

The MRI evaluation protocols used in the present study have been shown to have substantial to almost perfect reproducibility in relation to the evaluation of disc signal, disc height, and VESC with Kappa values for intra- and inter-observer reproducibility ranging from 0.77 to 1.0 and 0.72 to 0.91 respectively[19,20].

### Data analysis

An analysis was performed on people who participated in the study at both the age of 40 and 44 (responders) and those who only participated in the original study at age 40 (non-responders). The proportions of the following baseline variables were analysed at age 40: gender, presence of VESC, disc contour, disc degeneration, spondylolisthesis, LBP, heavy smoking, heavy physical workload, Body Mass Index, highest educational level, employment status, and the Back Beliefs Questionnaire score. The only significant difference between non-responders (n = 68) and those who participated in the follow-up study was their employment status. Nineteen percent of the non-responders were unemployed as compared with only seven percent among those who participated in the follow-up study.

Descriptive data were obtained for the VESC variables at both ages. The characteristics and distribution of VESC were analysed by frequency tables and cross-tabulations. Difference in prevalence of VESC at the two points of time was tested with McNemar's test of symmetry. The association between VESC and disc degeneration was analysed using logistic regression analysis. The change in type and size of VESC was analysed by cross-tabulations of the same variable obtained at age 40 and 44.

## Results

Three hundred and forty four people aged 44 years (161 men and 183 women), of the original cohort of 412 individuals aged 40 (83%), had an MRI at both time points and were therefore included in this study.

### Characteristics at baseline

At the age of 40, 133 (39%) of the 344 people had a total of 237 endplate signal changes (see Table 1). Of the observed VESC, the majority (88%) were of only one type, either type 1 or type 2, and two-thirds were of only one VESC size category.

**Table 1:** Persons with VESC

		**Type of VESC**				**Size of VESC**				
			
		**Only one type**				**Only one size**				
					
	**Persons with VESC**	**Type 1**	**Type 2**	**Type 3**	Two or more*	**EP only**	**<25%**	**25–50%**	**>50%**	Two or more*
**Age 40**	133	113	4	0	16	57	24	5	3	44
**Age 44**	170	120	13	0	37	42	38	11	5	74

* The numbers indicate individuals who have two or more  categories of type or size.

The table shows the number of persons with VESC (type and size) among 344 persons from the Danish general population who had an MRI at the age of 40 and 44.

The distribution of the type, size, and location of VESC at age 40 in relation to vertebral levels is shown in Additional file 1. There was no difference in the prevalence rates in relation to gender or the involvement of the upper and lower endplates (data not shown). Type 1 was the most prevalent VESC, accounting for 214 (90%) of the 237 signal changes. The majority of the signal changes, 159 (67%) of 237, were observed in the lower lumbar spine, at the L4-5 and L5-S1 disc levels. At the upper lumbar levels (L1-2, L2-3, and L3-4), the majority of the signal changes were observed only in the endplate and were located in the anterior part of the vertebra, whereas in the lower part of the lumbar spine, VESC was more likely to also extend further into the vertebra and to involve more than just the anterior part of the endplate.

**Figure 2:**
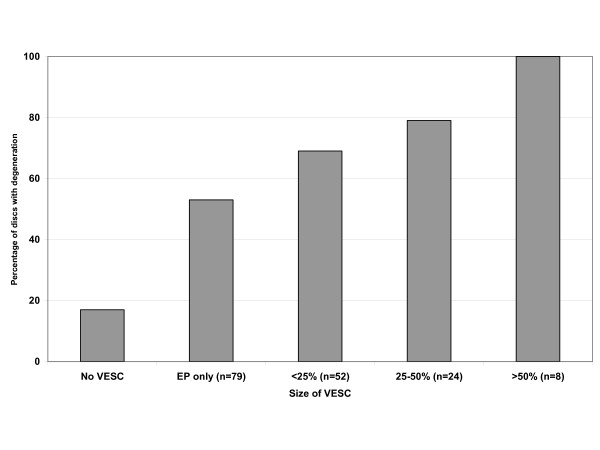
**VESC and disc degeneration**. The percentage of discs with degeneration (decreased signal or reduced height) in relation to the size of adjacent VESC.

Disc degeneration was present in 375 (22%) of 1720 discs evaluated at baseline. Disc degeneration was more common at disc levels with VESC (64%) than at levels without VESC (17%) p < 0.0001. Moreover, there was an association between disc degeneration and the size of VESC, as the proportion of discs with degeneration increased from 53% for VESC that was only observed in the endplate, to 100% for those that extended more than 50% into the vertebra, p < 0.0001 (see Figure 2). However, there was no statistically significant difference between the various types of VESC and disc degeneration (data not shown).

### Change of VESC over four years

**Figure 3:**
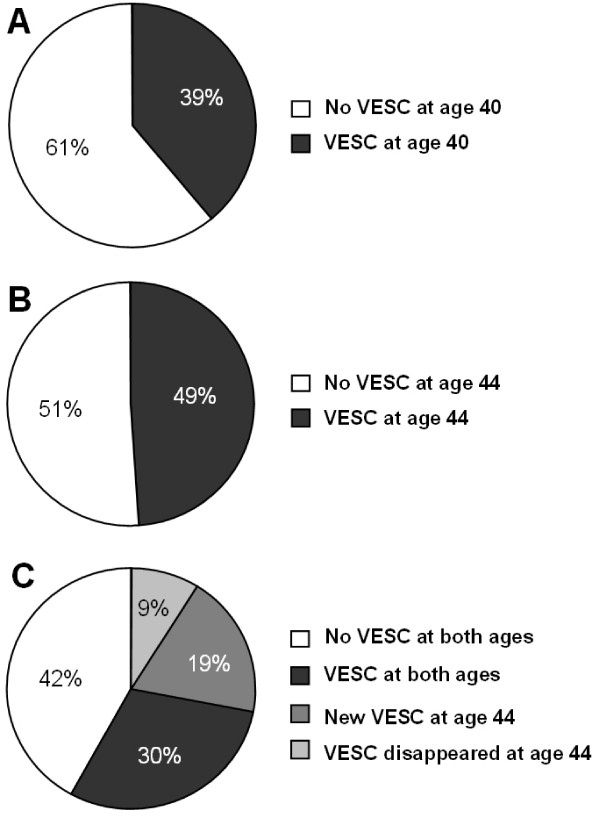
**The development of VESC**. The proportion of persons with VESC at age 40 (A) and age 44 (B) in 344 persons from the Danish general population. (C) represents the persons VESC status at age 44 in relation to their VESC status at age 40.

**Figure 4:**
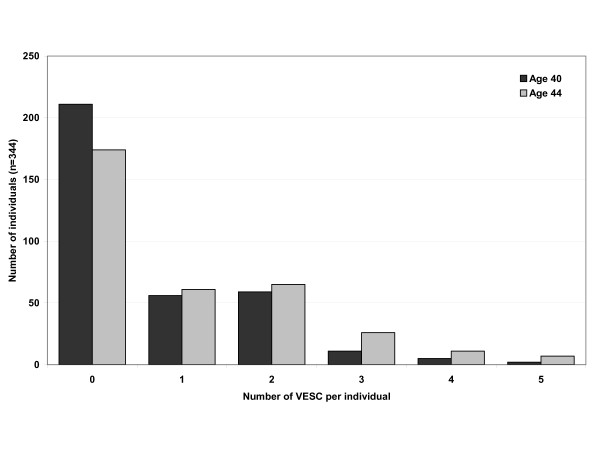
**VESC per person**. The number of VESC per person at age 40 and 44 in persons from the Danish general population (n = 344).

The prevalence of VESC increased significantly from age 40 to 44 for both the number of people with VESC and the number of vertebral levels involved (p < 0.0001). The number of people with VESC of any type or size, increased from 39% to 49% (see Figure 3). Moreover, the number of VESC per person also increased significantly (p < 0.0001, Wilcoxon matched-pairs signed-ranks test), see Figure 4. Of the 210 people who had no VESC at age 40, 67 had VESC four years later. One-hundred-and-three (30%) had VESC at both age 40 and 44. However, VESC is not necessarily permanent, as 23% of the 133 people who had VESC at age 40 years did not have these changes at age 44 years.

As mentioned above, the number of vertebral levels with VESC increased over the four-year period (see Additional files 1 and 2). Of the 3547 endplates that did not display VESC at age 40 years, six percent (195) had developed VESC at age 44. Sixty-five percent of the 237 endplates with VESC at age 40 persisted over the four-year period and the remaining 35% signal changes disappeared.

**Figure 5:**
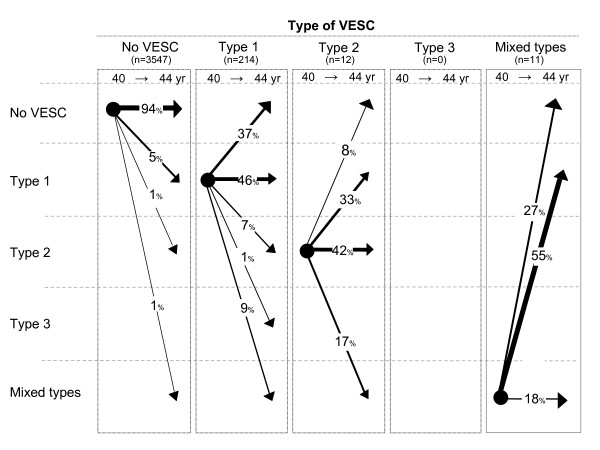
**The natural course of VESC types**. The natural course of VESC in relation to types of VESC in 3784 endplates from 344 persons from the Danish general population. Arrows indicate the change of VESC (in percent) from age 40 to age 44.

**Figure 6:**
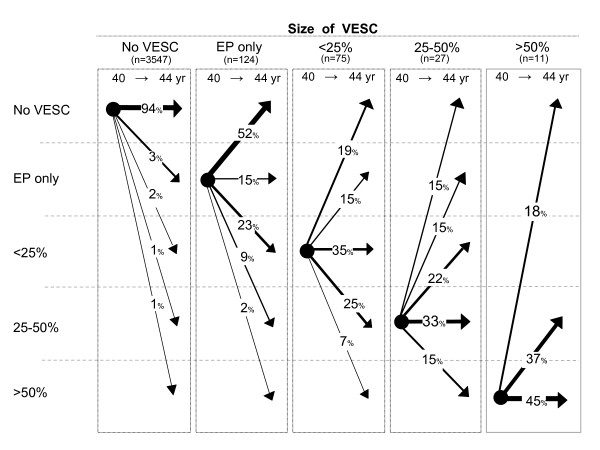
**The natural course of VESC size**. The natural course of VESC in relation to size of VESC in 3784 endplates from 344 persons from the Danish general population. Arrows indicate the change of VESC (in percent) from age 40 to age 44.

Of the 195 new VESC that appeared from age 40 to 44, the majority of these (85%) were type 1, the remainder being either type 2 or mixed type (see Figure 5). Furthermore, 48% of the new VESC was observed in the endplate only. Of the 153 endplates, in which VESC was detected at both baseline and follow-up, 32% changed type and 62% changed size over the four-year period. VESC that was observed in the endplate only was more likely to change over time than those that were larger (see Figure 6). The majority of the 84 signal changes that disappeared from baseline to follow-up were type 1 (n = 80) or located in the endplate only (n = 64) (see Table 2).

**Table 2:** New and disappeared VESC

	**Type of VESC** N(%)			**Size of VESC** N(%)			
		
	**Type 1**	**Type 2**	**Mixed types**	**EP only**	**<25%**	**25–50%**	**>50%**
**New VESC **(n = 195)	166 (85)	21 (11)	8 (4)	93 (48)	74 (38)	23 (12)	5 (3)
**Disappeared **(n = 84)	80 (95)	1 (1)	3 (3)	64 (76)	14 (17)	4 (5)	2 (2)

The table shows the proportions of type and size of VESC of new and disappeared signal changes from age 40 to age 44 (n = 84).

## Discussion

To our knowledge, this is the first study that has investigated the natural course of VESC in the general population. The results from this study show that: 1) the prevalence of VESC increases from the age of 40 to the age of 44 and 2) the distribution over vertebral levels, presence of disc degeneration and the natural course of VESC is dependent on the size of the signal changes.

The increase in the prevalence of VESC over a four-year period confirms previous observations that there is a positive association between age and the prevalence of VESC [3,4,9,10].

As stated in the introduction, other studies suggest that there is a difference in the size and distribution of VESC in patients with LBP compared with people without LBP[8,9,12]. In the current study, the association between size or location of VESC and the presence of pain was not investigated. However, VESC that was only observed in the endplate were equally distributed among the lumbar levels and often located in the anterior part of the endplate, whereas those extending beyond the endplate were found primarily at levels L4-L5 and L5-S1 and extended further along the endplate. Whether the size and location of VESC were associated with the LBP status of these people will be analysed and reported separately.

The positive association between the size of VESC and the presence of disc degeneration raises the question as to whether or not VESC is a response to advanced and/or accelerated disc degeneration. In support of this theory, two prospective studies of patients with sciatica treated non-surgically and surgically, have reported a large increase of type 1 changes over periods of 14 and 24 months respectively[6,7]. Further evidence in favour of this theory are results from studies on baboons and rats, which report that disc injury induces change in the adjacent vertebrae with subsequent bone marrow depletion, degeneration and regeneration of the bone [25-27].

Regarding the natural course of VESC, the results from this study show that the signal changes that were only observed in the endplate were those that tended to be transient, whereas those that extended beyond the endplate were more likely to persist over a four-year period. The most straightforward explanation for this is that the smaller the lesion, the easier it is for the body to heal itself. A related explanation could be that people in whom the VESC progress over time 1) are more prone to injury, 2) have a greater inflammatory response to injury, 3) have poorer regenerative abilities, and/or 4) have one or more of the lifestyle factors associated with VESC (i.e. smoking[2], hard work[2], and BMI[28]).

There are factors in the present study that could have influenced the reported prevalence of VESC and need to be addressed. The evaluation protocol used in this study described all VESC regardless of size[19], whereas the protocol from the previous study excluded the smallest VESC [20]. This might explain why the prevalence of VESC in people of 40 years of age reported in the present study was higher (39%) than previously reported (23%) for the same cohort[18]. Furthermore, the evaluation protocol used in the present study included four variables to evaluate the size of VESC (i.e. volume, maximum height, endplate area, and anteroposterior diameter). However, in daily clinical practice it would be impractical to use all four measures. Therefore, on the basis of personal communication with clinicians and researchers with various MRI experience, 'maximum height' was selected as the variable that was easiest to evaluate and was used as 'size' in the analysis. Furthermore, we did perform a sensitivity analysis using the three other variables and this did not change the results.

In relation to the high prevalence of type 1 changes, all MRI scans in the cohort study were performed on a low-field MRI unit (0.2 T). It is known that the contrast between different types of tissues are visualised differently at low-field scanners and high-field scanners. Therefore, it is possible that there are differences in the way that the different types of VESC are visualised on the two types of scanners. Results from a study of 20 patients with LBP and VESC, conducted at the Back Research Centre subsequent to data collection for the cohort study, showed that there are differences in the way that VESC is displayed on high- and low-field scanners[29]. When comparing high- and low-field MRIs from the same patients, the prevalence of VESC was 10% higher on high-field scanners as compared with low-field scanners. More importantly, the proportion of type 1 changes seen on low-field systems was three times greater than when evaluating the same patients on a high-field system. These results might explain why more than 90% of the signal changes at baseline were type 1 changes or mixed type 1 and 2 as compared with studies performed with high-field MRI systems, where the prevalence of VESC type 1 ranged between 3 and 50%[4,8-10,13,30-38].

The major strength of this study is that the study sample was population-based, representative of the Danish general population, and that all individuals were of the same age[18]. Furthermore, the protocols used in the study for the evaluation of VESC[19] and disc degeneration[20] have been shown to have excellent reproducibility when compared with previous studies using high-field systems [8,9,12,23,39-41].

## Conclusion

The results from this study indicate that the distribution of VESC, its association with disc degeneration and its natural course are dependent on VESC size.

## Competing interests

The authors declare that they have no competing interests.

## Authors' contributions

TSJ took part in all elements of the study and drafted the manuscript. TB contributed to the design of the study, data interpretation and revision of the manuscript. JSS contributed to the study design, data interpretation, and revision of the manuscript. LK contributed to the study design, participated in the data interpretation and supervised the statistical analyses. CM contributed to the design of the study and data interpretation. PK contributed to the design of the study, data interpretation, the statistical analyses, and revision of the manuscript. All authors read and approved the final manuscript.

## Pre-publication history

The pre-publication history for this paper can be accessed here:



## Supplementary Material

Additional file 1 **Characteristics of VESC at age 40**. Characteristics of VESC in relation to vertebral levels in 344 persons from the Danish general population at the age of 40. No type 3 changes were observed at age 40. Click here for file

Additional file 2 **Characteristics of VESC at age 44**. Characteristics of VESC in relation to vertebral levels in 344 persons from the Danish general population at the age of 44. Click here for file
