# Supplementary material for: Characteristics and natural course of vertebral endplate signal (Modic) changes in the Danish general population
Source: BMC Musculoskelet Disord. 2009 Jul 3;10:81. doi: 10.1186/1471-2474-10-81 (PMC2713204; doi:10.1186/1471-2474-10-81)
Supplement: Additional file 1 — Characteristics of VESC at age 40. Characteristics of VESC in relation to vertebral levels in 344 persons from the Danish general population at the age of 40. No type 3 changes were observed at age 40. [file 1471-2474-10-81-S1.doc]

**Table 2 – Characteristics of VESC at age 40**

Characteristics of VESC in relation to vertebral levels in 344 persons from the Danish general population at the age of 40. No type 3 changes were observed at age 40.

|  |  |  | **Type of VESC (N)** | | |  | **Size of VESC (N)** | | | |  | **Location of VESC (N)** | | | |
| --- | --- | --- | --- | --- | --- | --- | --- | --- | --- | --- | --- | --- | --- | --- | --- |
| **Vertebral level** | **No VESC** |  | **Type 1** | **Type 2** | **Mixed**  **type** |  | **EP only** | **<25%** | **25-50%** | **>50%** |  | **Central part only** | **Ant. part only** | **Post. part only** | **Two or**  **more*** |
|  |  |  |  |  |  |  |  |  |  |  |  |  |  |  |  |
| **L1 sup.** | 341 |  | 3 | 0 | 0 |  | 3 | 0 | 0 | 0 |  | 0 | 3 | 0 | 0 |
| **L1 inf.** | 337 |  | 6 | 0 | 1 |  | 6 | 1 | 0 | 0 |  | 1 | 4 | 1 | 1 |
|  |  |  |  |  |  |  |  |  |  |  |  |  |  |  |  |
| **L2 sup.** | 331 |  | 11 | 1 | 1 |  | 11 | 2 | 0 | 0 |  | 1 | 11 | 0 | 1 |
| **L2 inf.** | 333 |  | 10 | 0 | 1 |  | 6 | 3 | 2 | 0 |  | 1 | 6 | 1 | 3 |
|  |  |  |  |  |  |  |  |  |  |  |  |  |  |  |  |
| **L3 sup.** | 327 |  | 17 | 0 | 0 |  | 12 | 3 | 2 | 0 |  | 0 | 14 | 0 | 3 |
| **L3 inf.** | 334 |  | 8 | 1 | 1 |  | 6 | 2 | 1 | 1 |  | 2 | 3 | 3 | 2 |
|  |  |  |  |  |  |  |  |  |  |  |  |  |  |  |  |
| **L4 sup.** | 327 |  | 17 | 0 | 0 |  | 13 | 2 | 2 | 0 |  | 1 | 12 | 0 | 4 |
| **L4 inf.** | 310 |  | 29 | 1 | 4 |  | 13 | 11 | 6 | 4 |  | 6 | 8 | 3 | 17 |
|  |  |  |  |  |  |  |  |  |  |  |  |  |  |  |  |
| **L5 sup.** | 312 |  | 29 | 2 | 1 |  | 14 | 12 | 2 | 4 |  | 2 | 11 | 2 | 17 |
| **L5 inf.** | 288 |  | 48 | 6 | 2 |  | 17 | 25 | 12 | 2 |  | 1 | 9 | 4 | 42 |
|  |  |  |  |  |  |  |  |  |  |  |  |  |  |  |  |
| **S1 sup.** | 307 |  | 36 | 1 | 0 |  | 23 | 14 | 0 | 0 |  | 3 | 9 | 2 | 23 |
|  |  |  |  |  |  |  |  |  |  |  |  |  |  |  |  |
| **Total** | **3,547** |  | **214** | **12** | **11** |  | **124** | **75** | **27** | **11** |  | **18** | **90** | **16** | **113** |
|  | | | | | | | | | | | | | | | |

EP: endplate, Ant.: anterior, Post.: posterior

***** The numbers indicate endplates with VESC that extended over two or more locations
