# Supplementary material for: Characteristics and natural course of vertebral endplate signal (Modic) changes in the Danish general population
Source: BMC Musculoskelet Disord. 2009 Jul 3;10:81. doi: 10.1186/1471-2474-10-81 (PMC2713204; doi:10.1186/1471-2474-10-81)
Supplement: Additional file 2 — Characteristics of VESC at age 44. Characteristics of VESC in relation to vertebral levels in 344 persons from the Danish general population at the age of 44. [file 1471-2474-10-81-S2.doc]

**Table 3 – Characteristics of VESC at age 44**

Characteristics of VESC in relation to vertebral levels in 344 persons from the Danish general population at the age of 44.

|  |  |  | **Type of VESC (N)** | | | |  | **Size of VESC (N)** | | | |  | **Location of VESC (N)** | | | |
| --- | --- | --- | --- | --- | --- | --- | --- | --- | --- | --- | --- | --- | --- | --- | --- | --- |
| **Vertebral level** | **No VESC** |  | **Type 1** | **Type 2** | **Type 3** | **Mixed**  **type** |  | **EP only** | **<25%** | **25-50%** | **>50%** |  | **Central part only** | **Ant. part only** | **Post. part only** | **Two or**  **more*** |
|  |  |  |  |  |  |  |  |  |  |  |  |  |  |  |  |  |
| **L1 sup.** | 335 |  | 7 | 2 | 0 | 0 |  | 5 | 2 | 2 | 0 |  | 2 | 7 | 0 | 0 |
| **L1 inf.** | 335 |  | 8 | 0 | 0 | 1 |  | 7 | 1 | 1 | 0 |  | 2 | 6 | 0 | 1 |
|  |  |  |  |  |  |  |  |  |  |  |  |  |  |  |  |  |
| **L2 sup.** | 324 |  | 16 | 3 | 0 | 1 |  | 9 | 9 | 1 | 1 |  | 5 | 14 | 0 | 1 |
| **L2 inf.** | 323 |  | 18 | 2 | 0 | 1 |  | 11 | 8 | 2 | 0 |  | 2 | 12 | 1 | 6 |
|  |  |  |  |  |  |  |  |  |  |  |  |  |  |  |  |  |
| **L3 sup.** | 310 |  | 30 | 2 | 0 | 2 |  | 18 | 16 | 0 | 0 |  | 7 | 21 | 2 | 4 |
| **L3 inf.** | 324 |  | 15 | 1 | 0 | 4 |  | 9 | 10 | 1 | 0 |  | 8 | 4 | 5 | 4 |
|  |  |  |  |  |  |  |  |  |  |  |  |  |  |  |  |  |
| **L4 sup.** | 318 |  | 20 | 4 | 0 | 2 |  | 14 | 10 | 2 | 0 |  | 1 | 16 | 5 | 4 |
| **L4 inf.** | 296 |  | 30 | 9 | 1 | 8 |  | 10 | 23 | 12 | 3 |  | 4 | 9 | 6 | 29 |
|  |  |  |  |  |  |  |  |  |  |  |  |  |  |  |  |  |
| **L5 sup.** | 299 |  | 32 | 9 | 0 | 4 |  | 16 | 13 | 12 | 4 |  | 3 | 11 | 5 | 26 |
| **L5 inf.** | 276 |  | 52 | 8 | 0 | 8 |  | 12 | 22 | 23 | 11 |  | 12 | 7 | 3 | 46 |
|  |  |  |  |  |  |  |  |  |  |  |  |  |  |  |  |  |
| **S1 sup.** | 296 |  | 46 | 1 | 0 | 1 |  | 15 | 21 | 10 | 2 |  | 11 | 6 | 3 | 28 |
|  |  |  |  |  |  |  |  |  |  |  |  |  |  |  |  |  |
| **Total** | **3,436** |  | **274** | **41** | **1** | **32** |  | **126** | **135** | **66** | **21** |  | **57** | **113** | **30** | **148** |
|  | | | | | | | | | | | | | | | | |

EP: endplate, Ant.: anterior, Post.: posterior

***** The numbers indicate endplates with VESC that extended over two or more locations
